# Comparison of yield and relative costs of different screening algorithms for tuberculosis in active case-finding: a cross-section study

**DOI:** 10.1186/s12879-021-06486-w

**Published:** 2021-08-13

**Authors:** Fei Zhao, Canyou Zhang, Chongguang Yang, Yinyin Xia, Jin Xing, Guolong Zhang, Lin Xu, Xiaomeng Wang, Wei Lu, Jianwei Li, Feiying Liu, Dingwen Lin, Jianlin Wu, Xin Shen, Shuangyi Hou, Yanling Yu, Dongmei Hu, Chunyi Fu, Lixia Wang, Jun Cheng, Hui Zhang

**Affiliations:** 1 National Center for Tuberculosis Control and Prevention, Chinese Center for Disease Control and Prevention, Beijing, People’s Republic of China; 2 Clinical Trial Center, Beijing Hospital, National Center of Gerontology, Institute of Geriatric Medicine, Chinese Academy of Medical Sciences, Beijing, People’s Republic of China; 3 Department of Epidemiology of Microbial Diseases, Yale School of Public Health, New Haven, CT USA; 4 Institute of Tuberculosis Control and Prevention, Henan Provincial Center for Disease Control and Prevention, Zhengzhou, Henan People’s Republic of China; 5 Division of Tuberculosis Control and Prevention, Yunnan Provincial Center for Disease Control and Prevention, Kunming, Yunnan People’s Republic of China; 6 Institute of TB Control, Zhejiang Provincial Center for Disease Control and Prevention, Hangzhou, Zhejiang People’s Republic of China; 7 Department of Chronic Communicable Disease, Jiangsu Provincial Center for Disease Control and Prevention, Nanjing, Jiangsu People’s Republic of China; 8 Center for Tuberculosis Control of Guangdong Province, Guangzhou, Guangdong People’s Republic of China; 9 Guangxi Provincial Center for Disease Control and Prevention, Nanning, Guangxi Zhuang Autonomous Region People’s Republic of China; 10 Sichuan Provincial Center for Disease Control and Prevention, Chengdu, Sichuan People’s Republic of China; 11 Shanghai Municipal Center for Disease Control and Prevention, Shanghai, People’s Republic of China; 12 Hubei Provincial Center for Disease Control and Prevention, Wuhan, Hubei People’s Republic of China; 13 Heilongjiang Provincial Center for Tuberculosis Control and Prevention, Harbin, Heilongjiang People’s Republic of China; 14 Department of Emergency Medicine, Beijing Hospital, National Center of Gerontology, Institute of Geriatric Medicine, Chinese Academy of Medical Sciences, Beijing, People’s Republic of China

**Keywords:** Tuberculosis, Cost-effectiveness, Active case-finding, Screening

## Abstract

**Background:**

Part of tuberculosis (TB) patients were missed if symptomatic screening was based on the main TB likely symptoms. This study conducted to compare the yield and relative costs of different TB screening algorithms in active case-finding in the whole population in China.

**Methods:**

The study population was screened based on the TB likely symptoms through a face-to-face interview in selected 27 communities from 10 counties of 10 provinces in China. If the individuals had any of the enhanced TB likely symptoms, both chest X-ray and sputum tests were carried out for them furtherly. We used the McNemar test to analyze the difference in TB detection among four algorithms in active case-finding. Of four algorithms, two were from WHO recommendations including 1a/1c, one from China National Tuberculosis Program, and one from this study with the enhanced TB likely symptoms. Furthermore, a two-way ANOVA analysis was performed to analyze the cost difference in the performance of active case-finding adjusted by different demographic and health characteristics among different algorithms.

**Results:**

Algorithm with the enhanced TB likely symptoms defined in this study could increase the yield of TB detection in active case-finding, compared with algorithms recommended by WHO (p < 0.01, Kappa 95% CI: 0. 93–0.99) and China NTP (p = 0.03, Kappa 95% CI: 0.96–1.00). There was a significant difference in the total costs among different three algorithms WHO 1c/2/3 (F = 59.13, p < 0.01). No significant difference in the average costs for one active TB case screened and diagnosed through the process among Algorithms 1c/2/3 was evident (F = 2.78, p = 0.07). The average costs for one bacteriological positive case through algorithm WHO 1a was about two times as much as the costs for one active TB case through algorithms WHO 1c/2/3.

**Conclusions:**

Active case-finding based on the enhanced symptom screening is meaningful for TB case-finding and it could identify more active TB cases in time. The findings indicated that this enhanced screening approach cost more compared to algorithms recommend by WHO and China NTP, but the increased yield resulted in comparative costs per patient. And it cost much more that only smear/bacteriological-positive TB cases are screened in active case-finding.

**Supplementary Information:**

The online version contains supplementary material available at 10.1186/s12879-021-06486-w.

## Background

Tuberculosis (TB) remains an infectious disease imposing severe hazards to human health. Moreover, TB listed as public health problems to urgently be addressed today together with HIV/AIDS and malaria by the World Health Organization (WHO) and was one of the major infectious diseases under key control in China [1]. Although China has made significant achievements in TB prevention and control owing to the long-term efforts by the government and all-level health sectors, the current situation is not optimistic [2]. China is still one of 30 TB high-burden countries in the world, and 886 thousand estimated incidenct TB patients in China ranks second in the world [3].

The combination of case-finding and curing TB patients is widely recognized as the most cost-effectiveness measure for TB prevention and control [4, 5]. Due to limited resource, China currently adopts the strategy of passive case-finding and there are no more active case-finding measures except the symptom screening among the close contacts of pulmonary smear-positive TB patients and the individuals with HIV/AIDS [6]. The main TB likely symptoms include cough with expectoration ≥ 2 weeks, hemoptysis and bloody sputum based on the national TB control program in China (China NTP) [7]. However, the national TB prevalence survey in 2010 showed that if symptomatic screening for TB was implemented based on the major suspected symptoms defined by China NTP, part of TB patients could be missed [8, 9].

Previous studies have reported that any cough and other symptoms such as chest pain and loss of weight, had particular low sensitivity and specificity for TB detection [10, 11]. It meant that if the extended TB likely symptoms (such as chest pain) were applied as the criteria of further examinations for TB diagnosis, more cases would be detected. However, resource requirements for further tests may be prohibitive in some settings and a reason to opt for particular symptom screening [12].

Therefore, it was worthy to find a balance between the case detection and cost. We hypothesized that if more TB cases could be detected through the extended symptomatic screening compared with the general symptomatic screening strategies of WHO and China NTP. Here, we conduct this cross-section study in 10 provinces in China to compare the enhanced TB likely symptom screening algorithm in yield and cost of TB case finding with the screening algorithms from WHO and China NTP.

## Methods

### Ethics

The study protocol was approved by the Chinese center for disease control and prevention (China CDC) ethics committees (No. 201322). All participants before the enrollment signed the written informed consents. The written informed consent for the participants who were younger than 15 years old or patients with mental illness was obtained from their parent or guardian. All notified patients were referred to the local designated TB clinic or hospital for treatment according to national guidelines.

### Selection of study sites

We applied a multi-stage sampling to create a representative sample from the Chinese mainland, with the following steps:

First, nine provinces of 31 provinces in terms of the east, central and west China respectively, and one of the four municipalities directly under the Central Government (Beijing, Shanghai, Tianjin, and Chongqing) were selected according to the willingness to participate. These were Jiangsu province, Zhejiang province and Guangdong province in eastern China, Henan province, Heilongjiang province and Hubei province in central China, Sichuan province, Guangxi Zhuang autonomous region and Yunnan province in western China, and Shanghai city.

Then, one county/district that had more than 500,000 people, was selected simple-randomly in each selected province.

**Fig. 1:**
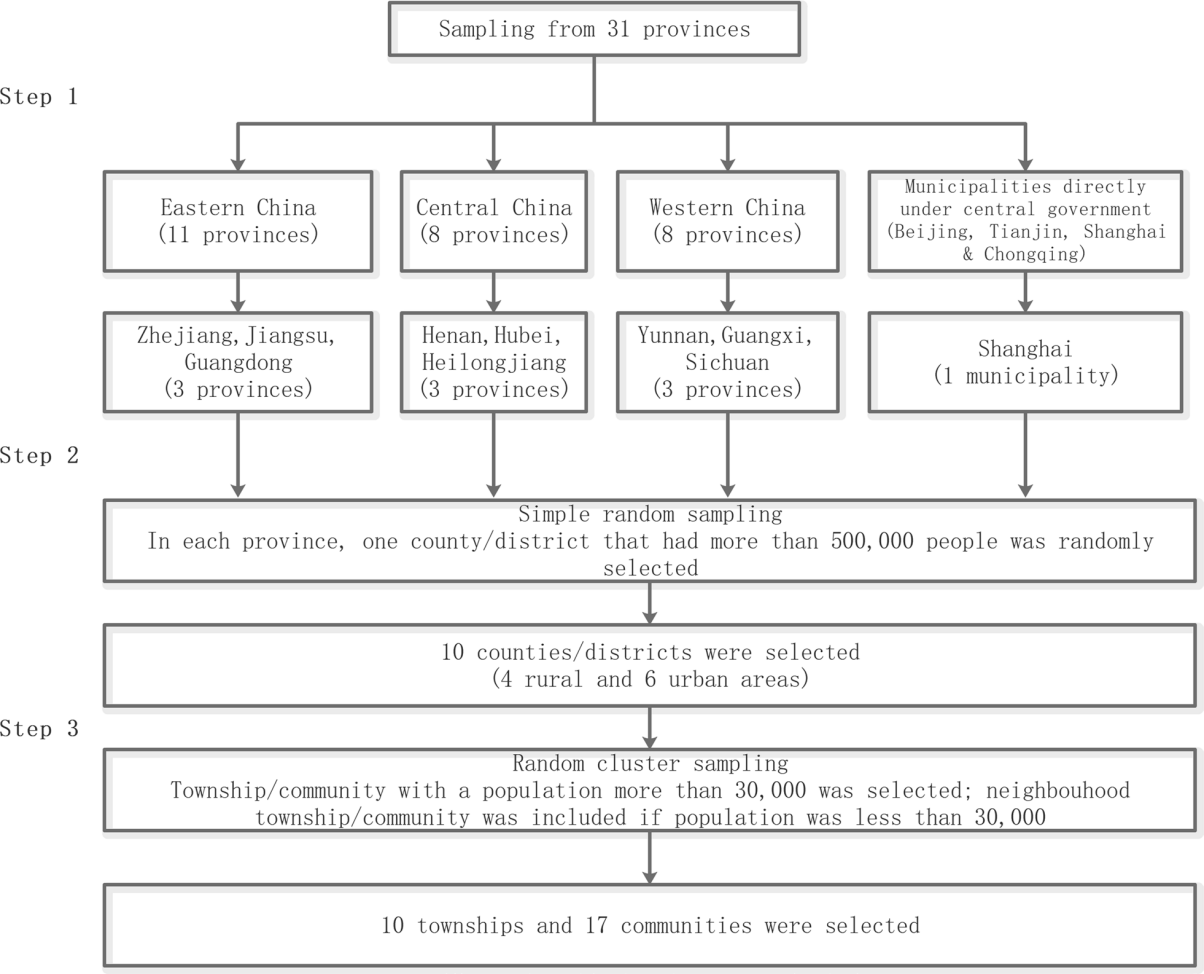
The sampling procedure of this study

Finally, the township/community was selected simple-randomly from each enrolled county. If the total number of the general population in selected township/community were less than 30,000 people, the neighborhood township/community would also be enrolled in the study site, till up to 30,000 people (Fig. 1) [13]. Totally ten townships and 17 communities were selected from 10 counties of 10 provinces/municipalities.

### Study population

The general population, who had been continuously living, working or studying in the survey sites for six months and above, including registered and non-registered population, were enrolled in the study. Totally 320,590 of the general population were included in the selected townships/communities [13].

### Field investigation and diagnosis

A face-to-face questionnaire-based inquiry was adopted to investigate whether the participant had any enhanced TB likely symptoms. The enhanced TB likely symptom was defined as any of the following conditions: 1) cough lasting longer than two weeks; 2) hemoptysis or bloody sputum; 3) cough longer than one week yet less than two weeks, and accompanied with any of the following symptoms: fever, night sweats, chest pain, loss of appetite, fatigue, or weight loss(> 3 kg).

Participants with any of the enhanced TB likely symptoms were offered chest X-ray (CXR) examination and requested to submit three sputum samples (morning, night, and spot sputum) for both sputum smear microscopy and culture test.

If participants < 15 years old had any enhanced TB likely symptoms, they were firstly supplied tuberculin skin test (PPD). Then, only those young participants with PPD induration ≥ 10 mm or blisters recieved CXR examination so that children and teenagers will not received unnecessary X-ray exposure.

Patients with smear-positive or culture-positive sputum were diagnosed as bacteriological positive TB. Patients with pulmonary tuberculosis (PTB) were defined as those with the bacteriological positive case and those diagnosed only by lesions on chest imaging known as clinically diagnosed cases.

The TB diagnosis group in each county composed of at least three health staffs, including a clinical doctor, a radiologist, and a laboratory technician, engaged in TB diagnosis. The criteria of TB diagnosis were compliance with the request of *Diagnostic Criteria for Tuberculosis in China* (WS288-2008) and the quality control was done according to the China National Guidelines [7].

### Data collection

In our study, the participants were investigated by a specially trained investigation group each county for data collection based on the questionnaire from July to September 2013. The specially trained investigation group included researchers, health-care workers, community workers, and local government staff. The participants were interviewed for any enhanced TB likely symptoms. At the same time, their sex, age, ethnicity, occupation, marital status, educational level, medical history, smoking and drinking habit, socioeconomic status and TB-related factors (Table 1) were collected as well. Moreover, their height and weight were measured to calculate BMI indicating their nutritional status.

**Table 1:** Definitions of terms of data collocation

Term	Definition
Sex	Male, Female
Age group	0 ~ , 15 ~ , 25 ~ , 35 ~ ,45 ~ , 55 ~ , 65 ~ ,75 ~ ,85 ~
Ethnicity	Han, other
Occupation	Unknown, Child/Student, Housework/Unemployment; Teacher, Service provider, Health-care worker, General worker, Farmer, Migrant worker, Retirement, Other
Marital status	Married, Single/divorced, Unknown
Education level	Unknown, Illiterate or semi-illiterate, Elementary school, Secondary school, College and above
Previous TB case	Registered in TB Information Management System, and finished treatment or cure
HIV/AIDS	Registered in the local CDC database, who were diagnosed according to diagnostic criteria for HIV/AIDS published by the National Health Commission of the People’s Republic of China in 2008
Known diabetes	Recorded on the Citizen Health Management Files as diagnosed with Diabetes (fasting plasma glucose level ≥ 7.0 mmol/L, or 2-h plasma glucose level ≥ 11.1 mmol/L), plus those using medicine to control blood glucose by self-report
Close contact	Living with a new active PTB case for at least 7 days in the 3 months before diagnosis
BMI level [14]	Underweight(BMI < 18.5), Normal(18.5 ≤ BMI < 24.0), Overweight(24.0 ≤ BMI < 28.0), fat(BMI ≥ 28.0)
Tobacco use	Ever smoked tobacco by self-report
Drinking habit	Drinking more than one unit(21 g pure alcohol) per week by self-report
Chronic bronchitis	Chronic bronchitis history by self-report
Average family annual income per capita	Average annual income per capita of urban family = RMB 27,000 (USD 3970), average annual income per capita of rural family = RMB 8000 (USD 1176), (USD 1 = RMB 6.8)
Average family living area per capita	Average of urban family = 29 m^2^, an average of rural family = 31 m^2^

All data on the questionnaire were entered in time and real-time double-checked by the online system developed especially for this study.

### TB screening algorithms with different paths

To evaluate the yield of different symptom combination, four algorithms were compared, including Algorithm 1a/1c from WHO, Algorithm 2 from China National TB Control Program, and Algorithm 3 from our study.

#### Algorithm WHO 1a

All people with a cough lasting longer than 2 weeks were investigated for TB. Sputum smear microscopy was considered as a second screening for people who have had a cough lasting longer than 2 weeks, and people with positive smear microscopy suggestive of TB should be diagnosed for TB. If sputum smear microscopy was negative and clinical suspicion was high, then considered further culture test for TB. The diagnosed TB cases were bacteriological positive cases. However, active TB cases who had negative sputum smear microscopy and the negative culture test could not be diagnosed due to a lack of chest X-ray.

#### Algorithm WHO 1c

Further investigation for TB was done for persons with a cough lasting longer than 2 weeks. Chest radiography was considered as a second screening for people who screened positive when asked about symptoms, and people with an abnormal chest radiograph suggestive of TB should be evaluated by sputum smear microscopy and culture test for TB. Therefore, active TB cases who had negative sputum smear microscopy and negative culture test could be diagnosed due to the performance of symptom screening and Chest X-ray.

#### Algorithm 2

the screening algorithm based on China NTP: People with a cough lasting longer than 2 weeks, hemoptysis, or bloody sputum were investigated further for TB. Chest radiography was considered as a second screening where chest X-ray is available for people who screened positive when asked about symptoms. People with suspected symptoms or an abnormal chest radiograph suggestive of TB should be evaluated by sputum smear microscopy and culture for TB. So active TB case with negative sputum smear microscopy and negative culture test could be diagnosed as well, beside bacteriological positive TB case.

#### Algorithm 3

(The enhanced symptoms screening algorithm for TB)**:** Persons with any of the following 3 options suggestive of TB should be further evaluated for TB. (1) cough lasting longer than 2 weeks; (2) hemoptysis or bloody sputum; (3) cough longer than 1 week yet less than 2 weeks, and accompanied with any of the following symptoms: fever, night sweats, chest pain, loss of appetite, fatigue, or weight loss(> 3 kg). Chest radiography was considered as a second screening for people who screened positive when asked about symptoms. People with suspected symptoms or an abnormal chest radiograph suggestive of TB should be evaluated by sputum smear microscopy and culture for TB. The TB diagnostic process was the same as Algorithm 2.

Algorithms WHO 1a/1c for screening and diagnosis were one of the recommendations from WHO [15–17]. Of 3 options of Algorithm 3, option (3) had been enhanced added to the definition of the TB likely symptoms in this study compared with Algorithm 2 the definition from China NTP. (Table 2) The participants had received all screening procedures in our study according to Algorithm 3, so the study population and case detection of algorithm WHO 1a/1c and 2 were extracted according to the definition of different algorithms.

**Table 2:** The screening procedure through algorithm WHO 1a/1c, 2, and 3

Algorithm	Household primary screening			Chest X-ray	Smear	Culture
	1)	2)	3)			
WHO 1a	✗				✗	✗
WHO 1c	✗			✗	✗	✗
2	✗	✗		✗	✗	✗
3	✗	✗	✗	✗	✗	✗

(1) cough lasting longer than two weeks; (2) hemoptysis or bloody sputum; (3) cough longer than one week yet less than two weeks, and accompanied with any of the following symptoms: fever, night sweats, chest pain, loss of appetite, fatigue, or weight loss(> 3 kg)

### Costs of active case screening

The cost was calculated among different screening algorithms based on the standard criteria of each component of the screening process, thereof 0.15 dollars per person-time for primary household screening by village health workers, 9.0 dollars per time for Chest X-ray, 3.9 dollars per time for sputum smear, 4.8 dollars per time for sputum culture[13]. Algorithm WHO 1a needed $8.85 for each person with suspected symptoms, and algorithm WHO 1c/2/3 needed $17.85. The functions of total cost of different algorithms were as followed:

Function 1:

$$Total cost of screening \left(Algorithm WHO 1a\right)=number of study population\times \$0.15+number of participatns with suspected symptom\times (\$3.9+\$4.8)$$

Function 2:

$$Total cost of screening \left(Algorithm WHO 1c/2/3\right)=number of study population\times \$0.15+number of participatns with suspected symptom\times (\$9+\$3.9+\$4.8)$$

### Statistical analysis

The prevalence of TB likely symptoms and TB cases was calculated. The McNemar test was used to analyze the difference in case detection between every two algorithms. Two-way ANOVA test was performed to evaluate the difference in the costs of different algorithms adjusted by demographic and health characteristics. Two-sided *p* < 0.05 was considered to be significant. All tests were performed using SAS 9.4 (SAS Institute, Cary, NC, USA).

## Results

### Demographic and health characteristics

There was a total of 320,590 eligible population, of which 299,610 persons (93.5%) were enrolled in the survey. Inaddtion to the questionnaire investigation, the CXR examination were performed for the people with the enhanced suspected symptoms. Males accounted for 50.2% (150,461), and females for 49.8% (149,149). The mean age was 39-year-old (median 40-year-old) Table 3.

**Table 3:** Demographic characteristics of the enrolled population in China in 2013

Variable	Number	Proportion(%)
All	299,610	100.00
Sex		
Male	150,461	50.2
Female	149,149	49.8
Age group		
0 ~	51,537	17.2
15 ~	32,312	10.8
25 ~	40,259	13.4
35 ~	48,363	16.1
45 ~	47,675	15.9
55 ~	45,195	15.1
65 ~	21,685	7.2
75 ~	10,658	3.6
85 ~	19,26	0.6
Ethnicity		
Han	268,145	89.5
Other	31,465	10.5
Occupation		
Unknown	13,757	4.6
Child/Student	63,867	21.3
Housework/Unemployment	17,627	5.9
Teacher	3103	1.0
Service provider	6132	2.1
Health-care worker	1155	0.4
General worker	37,639	12.6
Farmer	104,926	35.0
Migrant worker	8951	3.0
Retirement	19,999	6.7
Other	22,454	7.5
Marital status		
Married	190,635	63.6
Single/divorced	99,173	33.1
Unknown	9802	3.3
Education level		
Unknown,	10,491	3.5
Illiterate or semi-illiterate,	37,016	12.4
Elementary school,	77,294	25.8
Secondary school,	146,082	48.8
College and above	28,727	9.6
Previous TB case		
Yes	1595	0.5
No	298,015	99.5
HIV/AIDS		
Yes	59	< 0.1
No	299,551	> 99.9
Known diabetes		
Yes	5150	1.7
No	294,460	98.3
Close contact		
Yes	378	0.1
No	299,232	99.9
BMI level		
< 18.5	18,031	6.0
18.5 ≤ BMI < 24.0	160,854	53.7
24.0 ≤ BMI < 28.0	57,858	19.3
BMI ≥ 28.0	11,457	3.8
Unknown	51,410	17.2
Tobacco use		
Never smoke	196,412	65.6
Prior smoker	4948	1.7
Current smoker	46,854	15.6
Unknown	51,396	17.2
Drinking habit		
Never drink	197,481	65.9
Prior alcohol user	5,107	1.7
Current alcohol user	45,348	15.1
Unknown	51,674	17.3
Chronic bronchitis		
Yes	3381	1.1
No	244,041	81.5
Unknown	52,188	17.42
Pneumoconiosis		
Yes	178	< 0.1
No	247,993	82.8
Unknown	51,439	17.2
Average family annual income per capita*		
Higher than average	91,203	33.0
Lower than average	185,220	67.0
Average family living area per capita*		
Higher than average	105,812	38.2
Lower than average	171,007	61.8

Note: * Missing values existed

### Comparison in case detection between each two algorithms

There were significant differences in the case detection of active TB cases in the comparisons between Algorithm WHO 1c and 3 (p < 0.01, Kappa 95%CI: 0. 93–0.99) and between Algorithms 2 and 3 (p = 0.03, Kappa 95% CI: 0.96–1.00). No significant difference was observed between any other two algorithms in the same type of TB detection. (Table 4).

**Table 4:** McNemar’s test for detection rate between different symptom screening algorithms, p-value (95%CI for Kappa)

Algorithm	Type of patient	Type of patient in different algorithms					
		2	2	2	3	3	3
		Smear +	Bact +	Active TB	Smear +	Bact +	Active TB
WHO 1a	Smear +	0.32 (0.95,1.00)	–	–	0.05 (0.86,1.00)	-	–
WHO 1a	Bact +	-	0.32 (0.96,1.00)	–	–	0.05 (0.90,1.00)	–
WHO 1c	Smear +	0.32 (0.95,1.00)	-	–	0.05 (0.86,1.00)	–	–
WHO 1c	Bact +	–	0.32 (0.96,1.00)	–	–	0.05 (0.90,1.00)	–
WHO 1c	Active TB	–	–	0.08 (0.97,1.00)	–	–	< 0.01 (0.93,0.99)
2	Smear +	–	–	–	0.08 (0.89,1,00)	–	–
2	Bact +	–	–	–	–	0.08 (0.92,1.00)	–
2	Active TB	–	–	–	–	–	0.03 (0.96,1.00)

No inconsistency in the TB detection rate of sputum smear-positive and bacteriological positive between algorithm WHO 1a/1c

### The yield of different algorithms and their costs

Table 5 summarized the numbers of people screened in this study via different algorithms, the corresponding numbers of people with suspected symptoms, active tuberculosis cases diagnosed, and the related costs.

**Table 5:** Yield and relative costs of different symptom screening algorithms

Type of patient	Algorithm	Symptom No	No. of TB case	TB detection and screening		Relative costs	
				Detection proportion*(%)	Screening No. per case	Total costs	Average costs for one case
Smear +	1a	2328	27	1.16	11,097	65,195.1	2414.6
	1c	2328	27	1.16	11,097	86,147.1	3190.6
	2	2407	28	1.16	10,700	87,545.4	3126.6
	3	2676	31	1.16	9665	92,306.7	2977.6
Bact +	1a	2328	37	1.59	8098	65,195.1	1762.0
	1c	2328	37	1.59	8098	86,147.1	2328.3
	2	2407	38	1.58	7884	87,545.4	2303.8
	3	2676	41	1.53	7308	92,306.7	2251.4
Active TB	1c	2328	105	4.51	2853	86,147.1	820.4
	2	2407	108	4.49	2774	87,545.4	810.6
	3	2676	113	4.22	2651	92,306.7	816.9

*Detection proportion among people with suspected symptoms

In this study, both Algorithm 1c and Algorithm 2 notified one active TB case per average 22 (2328/105, 2407/108) persons with TB likely symptoms screened. Compared with algorithm WHO 1c, Algorithm 3 identified 8 (113–105) more active TB cases among an additional 348 (2676–2328) persons screened with the option 3) (cough longer than 1 week yet less than 2 weeks, and accompanied with any of the following symptoms: fever, night sweats, chest pain, loss of appetite, fatigue, or weight loss (> 3 kg)), which indicated an additional average of 44 (348/8) persons to be screened to diagnose one more active TB case. Meanwhile, Algorithm 3 required an additionally screening of 269 (2676–2407) persons with the option 3) compared to the Algorithm 2 for the diagnosis of 5 (113–108) more active TB cases. It suggested that an average of 54 (269/5) persons with option 3) of the enhanced TB likely symptoms screening beyond the TB likely symptoms defined by Algorithm 2 needed to be screened further to detect one more active TB case through Algorithm 3. When Algorithm 2 based on current China NTP was considered as “Golden standard”, 1) through Algorithm 3, the false positive rate was 1.7/100,000 and false negative rate was 0; 2) through Algorithm WHO 1c, the false positive rate 0 and false negative rate was 2.8%. Because Algorithm WHO 1a did not conduct CXR test as one of screening procedure, so it was not reasonable to compare between Algorithm 2 and WHO 1A.

Of 299,610 persons, 2328 with the TB likely symptoms defined by Algorithm WHO 1a/1c, were identified. Therefore, 37 bacteriological-positive TB cases via algorithm WHO 1a were diagnosed and the total costs of the processes of Algorithm WHO 1a were $65,195.1, which meant it cost $1762.0 per bacteriological-positive TB case diagnosed by Algorithm WHO 1a. There were as many as the persons with the TB likely symptoms identified and 105 active TB cases diagnosed via algorithm WHO 1c. The total costs of Algorithm WHO 1c were $86,147.1, which indicated that it cost $820.4 per active TB case diagnosed by algorithm WHO 1c. Additionally, algorithm 2 cost $810.6 ($87,545.4/108) for one active TB case, among 108 active TB cases from 2407 persons with suspected symptoms. Finally, the cost of $816.9 ($92,306.7/113) for one active TB case was estimated among 113 active TB cases from 2676 persons with suspected symptoms defined by algorithm 3.

**Fig. 2:**
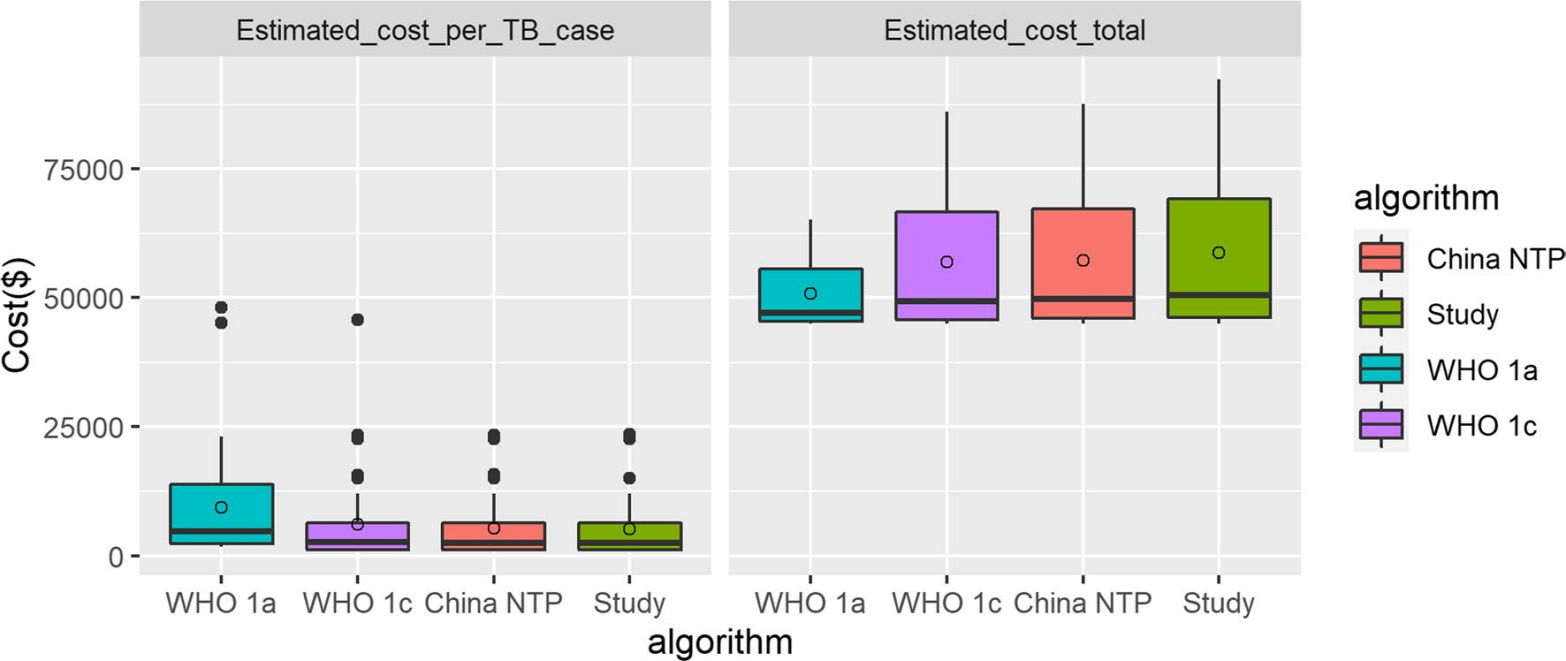
The comparison of 4 algorithms in total costs and average costs for one TB case. The cases all diagnosed through Algorithm WHO 1a were bacteriological positive; the cases diagnosed through Algorithms WHO 1c/2/3 were active TB cases.

A two-way ANOVA adjusted by different TB types and screening algorithms revealed that there was a significant difference in the total costs of screening for active TB cases in different Algorithms WHO 1c/2/3 (F = 59.13, p < 0.01, Additional file 1: Appendix S1). Both the number of TB cases and the total costs through algorithm 3 were maximum, compared with Algorithms 1c/2. However, no significant difference was evident in the average costs for one active TB case screened and diagnosed through the process among Algorithms WHO 1c/2/3 (F = 2.78, p = 0.07, Additional file 1: Appendix S1). On the other hand, the average costs for one bacteriological positive case through Algorithm WHO 1a was about two times as much as the costs for one active TB case through Algorithms WHO 1c/2/3 (Fig. 2).

## Discussion

Compared with WHO recommendation and China NTP guideline, more persons with TB likely symptoms were identified through Algorithm 3, and the total costs of this algorithm increased significantly as well. Furthermore, the introduction of the additional enhanced TB likely symptoms induced more active TB cases diagnosed. However, the average costs for one active TB case diagnosis through Algorithm 3 were no signs of a difference compared with Algorithms WHO 1c and 2. While much more costs should spend if the screening targeted at a bacteriological positive TB case.

During the past few years, there has been an intensified discussion about using active case-finding, or screening, as a possible complement to the predominant approach of “passive case-finding”. The primary objective of screening is to ensure that active TB is detected early to reduce the risk of poor disease outcomes and the adverse social and economic consequences of the disease, as well as help reduce TB transmission [16]. Although when a country is striving to eliminate TB and needs to invest additional resources to effectively reach those who are hardest to reach, active screening may be a crucial part of the response to TB [18]. Besides, the alternative screening algorithms and their costs should be considered based on sufficient evidence as well, especially in recourse-constrained high-burden countries [19]. In our study, the additional enhanced suspected symptoms during the screening could identified more active TB. And this algorithm in the costs for one active TB case was comparable to the algorithms of WHO/China NTP. Thus, it will be of interest to take active TB screening potentially in the low TB burden countries with sufficient resources or the resource-constrained countries striving to eliminate TB. The enhanced suspected symptoms for active TB screening was an alternative and could identify more active TB cases to reduce TB transmission. However, that was undoubtedly low cost-effectiveness for only screening smear/bacteriological positive TB cases in the active case-finding or screening. Our study indicated that the average costs for detecting one bacteriological positive case through Algorithm WHO 1a were approximately double as the costs for detecting one active TB case through Algorithms WHO 1c/2/3.

The costs of active case-finding appears to be different in a variety of settings [20]. The costs of active case-finding were more than passive case-finding [21]. However, the enhanced TB suspected symptoms in our study could help diagnose more active TB cases sooner, which was similar to that found in comparable studies [22]. Furthermore, compared with other studies in active screening for TB, our average screening costs for one active TB case was less than the same study in Russian Federation, despite the same level of TB incidence in both countries, but more than the studies in the higher TB-incidence countries [3, 21, 23, 24]. In addition, if the active case-finding might be performed in targeted high-risk groups, the costs of performance could decrease undoubtedly.

Our study is population-based research. These findings provided insights into the yield and costs of different active case-finding approaches. Our research groups had tried the best to keep the quality control. Although the large sample size allowed further analysis in different demographic and health characteristics, some inevitable missing values, like other extensive population studies, made these findings accompany with uncontrolled little information bias. And there might be false positives including particularly as 64% of the diagnoses through Algorithm 3 were not confirmed by smear or culture.

## Conclusions

Active case-finding based on the enhanced symptom screening is meaningful for TB case-finding and it could identify more active TB cases in time. The findings indicated that this active case-finding cost more into enhanced screening but the increased yield of active TB cases resulted in comparative costs per patient, despite increased total costs. This active case-finding potentially in the low TB burden countries with sufficient resources or the resource-constrained countries striving to eliminate TB as an alternative could identify more active TB cases to reduce TB transmission. And it cost much more that only smear/bacteriological positive TB cases are screened in active case-finding.

## Supplementary Information

**Additional file 1.** Results of Symptom screening and TB detection in different algorithms and variables..

## Data Availability

The National Center for Tuberculosis Control and Prevention (NCTB) is the custodian of the data for this study. The data are not accessible online but may be made available upon written request to the NCTB through the authors, if in line with the Ethical Review Board guidelines.

## Abbreviations

ACF: Active case finding
AIDS: Acquired immune deficiency syndrome
BMI: Body mass index
CI: Confidence interval
CXR: Chest X-ray
HIV: Human immunodeficiency virus
PTB: Pulmonary tuberculosis
TB: Tuberculosis
